# Early Renal Vasodilator and Hypotensive Action of Epoxyeicosatrienoic Acid Analog (EET-A) and 20-HETE Receptor Blocker (AAA) in Spontaneously Hypertensive Rats

**DOI:** 10.3389/fphys.2021.622882

**Published:** 2021-01-28

**Authors:** Agnieszka Walkowska, Luděk Červenka, John D. Imig, John R. Falck, Janusz Sadowski, Elżbieta Kompanowska-Jezierska

**Affiliations:** ^1^Department of Renal and Body Fluid Physiology, Mossakowski Medical Research Institute, Polish Academy of Sciences, Warsaw, Poland; ^2^Center for Experimental Medicine, Institute for Clinical and Experimental Medicine, Prague, Czechia; ^3^Department of Pathophysiology, 2nd Faculty of Medicine, Charles University, Prague, Czechia; ^4^Department of Pharmacology and Toxicology, Medical College of Wisconsin, Milwaukee, WI, United States; ^5^Department of Biochemistry, University of Texas Southwestern Medical Center, Dallas, TX, United States

**Keywords:** hypertension, 20-HETE antagonist, EET analog, soluble epoxide hydrolase, epoxyeicosatrienoic acids

## Abstract

Cytochrome P450 (CYP-450) metabolites of arachidonic acid: epoxyeicosatrienoic acids (EETs) and 20-hydroxyeicosatetraenoic acid (20-HETE) have established role in regulation of blood pressure (BP) and kidney function. EETs deficiency and increased renal formation of 20-HETE contribute to hypertension in spontaneously hypertensive rats (SHR). We explored the effects of 14,15-EET analog (EET-A) and of 20-HETE receptor blocker (AAA) on BP and kidney function in this model. In anesthetized SHR the responses were determined of mean arterial blood pressure (MABP), total renal (RBF), and cortical (CBF) and inner-medullary blood flows, glomerular filtration rate and renal excretion, to EET-A, 5 mg/kg, infused i.v. for 1 h to rats untreated or after blockade of endogenous EETs degradation with an inhibitor (*c*-AUCB) of soluble epoxide hydrolase. Also examined were the responses to AAA (10 mg/kg/h), given alone or together with EET-A. EET-A significantly increased RBF and CBF (+30% and 26%, respectively), seen already within first 30 min of infusion. The greatest increases in RBF and CBF (by about 40%) were seen after AAA, similar when given alone or combined with EET-A. MABP decreased after EET-A or AAA but not significantly after the combination thereof. In all groups, RBF, and CBF increases preceded the decrease in MABP. We found that in SHR both EET-A and AAA induced renal vasodilation but, unexpectedly, no additive effect was seen. We suggest that both agents have a definite therapeutic potential and deserve further experimental and clinical testing aimed at introduction of novel antihypertensive therapy.

## Introduction

Cytochrome P450 (CYP-450) metabolites of arachidonic acid, generated mostly by the vascular endothelium and smooth muscle cells, display distinct vasoactivity and have established role in the control of blood pressure (BP) and kidney function. The major metabolite group, epoxyeicosatrienoic acids (EETs), exhibit anti-hypertensive properties that depend not only on their vasodilator but also on the natriuretic potency (Imig, 2013, 2019). Another CYP-450 metabolite, 20-hydroxyeicosatetraenoic acid (20-HETE), is known for its dual action: it is both a prohypertensive agent (constricts the renal preglomerular microvasculature), and has antihypertensive properties (inhibits renal tubular sodium reabsorption) (Roman, 2002; Roman and Fan, 2018).

Epoxyeicosatrienoic acids are biologically unstable as they are rapidly metabolized by soluble epoxide hydrolase (sEH) to inactive dihydroxyeicosatrienoic acids (DiHETEs). Therefore, the bioavailability of EETs can be increased or restored by pharmacologic sEH blockade (Neckář et al., 2012; Kujal et al., 2014). However, sEH inhibitors effectively increased tissue EETs, decreased BP and provided organ protection only under conditions of EETs deficiency, e.g., resulting from increased conversion of EETs to DHETEs dependent on enhanced activity of sEH (Honetschlägerová et al., 2011; Neckář et al., 2012). Another approach to increase EET activity is to apply agonistic analogs that were designed to reduce EETs degradation and improve their solubility (Imig et al., 2010).

While generation of EETs in SHR is increased compared to normotensive Wistar-Kyoto (WKY) rats (Yu et al., 2000), their plasma levels are reduced (Jiang et al., 2011). Increased sEH activity in SHR was reported to contribute to the development of hypertension (Fornage et al., 2002), and SHR were shown to have elevated sEH mRNA and protein level in the kidney cortex compared to WKY (Koeners et al., 2011), which results in increased conversion of EETs to inactive metabolites, at least in the kidney.

One approach to augment EETs activity is to inhibit their degradation which can be accomplished using a specific sEH inhibitor, cis-4-[4-(3-adamantan-1-yl-ureido)-cyclohexyloxy]-benzoic acid (*c*-AUCB). The other approach is administration of EET-A [disodium (S)-2-(13-(3-pentyl)ureido)-tridec-8(Z)-enamido) succinate], a stable analog of native 14,15-EET, the one with well-established biological activity, as documented in our previous studies (Jíchová et al., 2016; Červenka et al., 2018).

Beside the influence of EETs, increased renal formation of 20-HETE can also contribute to the development of hypertension, therefore inhibition of its renal production or blockade of its receptors could reduce hypertension in SHR. It was confirmed that 20-HETE plasma levels are higher in SHR than in WKY controls (Sacerdoti et al., 1989).

So far, there are not many studies confirming that a lack of EETs or an increased production of 20-HETE in SHR may be one of the factors causing high BP or lead to kidney damage. Previously, we demonstrated that chronic administration EET-A and AAA to adult SHR lowered BP and improved renal hemodynamics and function (Gawryś et al., 2020). However, effects of manipulating the two involved AA pathways must be complex and the possible time-dependence of the effects is unknown. Therefore, the first goal of the present study was to explore the role of the two active AA metabolites in the control of BP and renal hemodynamics and excretion in acute experiments, within a 2-h interval. An EET analog (EET-A) was applied in rats with or without pretreatment with *c*-AUCB, an inhibitor of sEH. In addition, we tested the effects of AAA (N-disodium succinate-20-hydroxyeicosa-6(Z),15(Z)-diencarboxamide), an antagonist of 20-HETE receptors, with the compound given alone or together with EET-A. The earlier chronic study showed that the effects of the two active agents were clearly additive. Therefore, the second goal here was to examine if this was or was not the case in a short-time acute investigation. An advantage of such acute studies was that they enabled detailed exploration of intrarenal hemodynamics.

## Materials and Methods

All the animal experiments were performed in the Department of Renal and Body Fluid Physiology, Mossakowski Medical Research Institute, Polish Academy of Sciences, Warsaw, Poland. The experimental procedures were approved by the First Ethical Committee for Animal Experimentation, Warsaw, which follows the European Directive 2010/63/EU on the protection of animals used for scientific purposes.

Male spontaneously hypertensive rats (SHR) were used, aged 16 weeks (established hypertension), weighing 293–333 g. The animals were housed in groups of 2–4, under 12:12-h light-dark cycle, and had free access to tap water and standard rat chow (dry pellets with 0.25% Na w/w, SSINFF GmbH, Soest, Germany). All measurements were performed between 11:00 a.m. and 2:00 p.m.

Acute experiments were designed to examine effects on mean arterial pressure (MABP), renal hemodynamics and excretion of an EET analog (EET-A), and of AAA, an antagonist of 20-HETE receptors, given separately or combined (Series I). In addition, effects of EET-A were tested in rats chronically pretreated with *c*-AUCB, an inhibitor of sEH (series II).

### Series I

#### Surgical Preparations

Rats were anesthetized with 100 mg/kg of intraperitoneal sodium thiopental (Sandoz GmbH, Kundl, Austria). Their body temperature was maintained at ∼37°C by application of a heated surgery table. A tracheal tube ensured free airways. The femoral vein was cannulated for infusion of fluids; during surgery, 3% bovine serum albumin solution was infused at 10 ml kg/h to sustain plasma volume. After completed surgery the albumin solution was replaced by one enabling measurement of inulin clearance i.e., glomerular filtration rate (GFR). A priming infusion of 3% inulin (Sigma Aldrich) in saline was first given, followed by maintenance infusion of 1.5% inulin (Roszkowska-Chojecka et al., 2015).

The femoral artery was cannulated for MABP measurement (Stoelting blood pressure system, Wood Dale, Illinois, United States) and the carotid artery for blood sampling.

The left kidney was exposed from a subcostal flank incision and immobilized in a plastic holder. The ureter was cannulated for timed urine collections. A non-cannulating renal artery Transonic probe connected with a Transonic flowmeter (type T106 Transonic System, Ithaca, NY, United States) was used to measure total renal blood flow (RBF).

Blood perfusion of different renal zones was measured as laser-Doppler fluxes using a laser-Doppler Periflux 4001 system (Perimed AB, Jarfalla, Sweden). The cortical blood flow (CBF) was measured using a probe placed on the kidney surface, and inner medullary blood flow (IMBF) using a needle probe inserted into the kidney to the depth 5 mm. The details of this measurement technique were described previously (Walkowska et al., 2015).

#### Protocols

Post-surgery control parameters were first recorded during a 30-min urine collection. Thereafter, 14,15-EET analog, disodium (S)-2-(13-(3-pentyl)ureido)-tridec-8(Z)-enamido)succinate (EET-A; 5 mg/kg) or N-disodium succinate-20-hydroxyeicosa-6(Z),15(Z)-diencarboxamide (AAA;10 mg/kg) were given, alone or combined. The dose of EET-A was determined in preliminary experiments and the AAA dose was validated earlier (Sedláková et al., 2018).

Both substances were infused i.v. during 1 h while two 30-min urine collections were made, followed by two 30-min recovery periods. Blood samples for inulin determination were withdrawn as needed. An additional untreated group was also studied as time-control (the agent’s saline solvent). After completed experiments the animals were euthanised by an intravenous overdose of sodium thiopenthal.

The animals were randomly assigned to the following groups:

1. EET-A i.v. (*n* = 7).
2. AAA i.v. (*n* = 7).
3. EET-A + AAA i.v. (*n* = 7).
4. Untreated (0.9% saline solvent i.v., *n* = 7).

### Series II

In two groups, the above basic protocol was preceded by 14 days’ pretreatment with cis-4-[4-(3-adamantan-1-yl-ureido)-cyclohexyloxy]-benzoic acid (*c*-AUCB) a sEH inhibitor (Hwang et al., 2007) or its solvent. As described earlier, *c*-AUCB was prepared fresh three times a week and given in drinking water at a concentration of 13 mg/l which provided 8.5 mg/kg/day (Sporková et al., 2014). For preparation of the infusate the crystalline *c*-AUCB (13 mg) was dissolved under 5-min sonification in ethanol (5 ml) and cyclodextrin (150 mg); this solution was added to 1 L water (Neckář et al., 2012).

Following surgical preparations as described for Series I, a protocol analogous with that for EET-A studies was applied.

The following groups were studied:

1. Pretreatment with *c*-AUCB in drinking water + EET-A i.v. (*n* = 7).
2. Pretreatment with *c*-AUCB solvent + EET-A i.v. (*n* = 7).

### Analytical Procedures

Urinary sodium and potassium were measured by flame photometry (Jenway PFP7, Dunmow, Essex, United Kingdom), and urine osmolality using a cryoscopic osmometer (Osmomat 030; Gonotec GmbH, Berlin, Germany). To measure GFR, the rapid microtiter plate assay was used for determination of inulin in rat plasma and urine. Plasma and urine samples were prepared by addition of indole-3-acetic acid (Sigma-Aldrich) and HCl and vortexing. After incubation, each sample was transferred to a 96-well microtiter plate and read spectrophotometrically at 490 nm using Omega FLUOstar apparatus (BMG Labtech GmbH, Ortenberg, Germany).

### Statistics

Data are expressed as means ± SEM. To define the *n* number needed to arrive at reliable determination of significant differences, appropriate power analysis was first performed. In general, the data were analyzed by repeated-measures ANOVA with a Bonferroni test in case of multiple comparisons. *P* < 0.05 was considered as indicating significance. For within one group or between two groups comparison, two-tailed Student’s *t* test for paired or unpaired samples, respectively, was applied. With more than two data sets or groups, the significance of changes was evaluated by multivariable ANOVA with repeated measurements, followed by Tukey’s *post hoc* test (STATISTICA, version 10.0; StatSoft Inc. Kraków, Poland).

## Results

In preliminary studies we examined the effect of administering different doses of EET-A (2.5, 5, and 10 mg/kg for 1 h) on MABP, renal hemodynamics and excretion in normotensive rats. None of the doses used caused changes in the parameters tested (data not shown). Hence, no regular experiments with WKY have been carried out, in accordance with Russell and Burch’s 3Rs (in compliance with the ARRIVE by NC3R guidelines) and the laws of Directive 2010/63/EU and the Act on the protection of animals used for scientific and educational purposes.

### Series I

**FIGURE 1:**
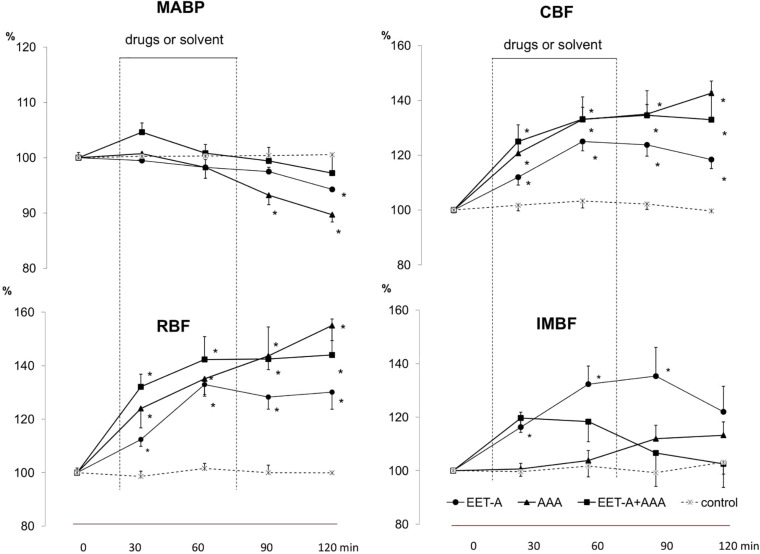
Effects of treatments (EET-A, 5 mg/kg; AAA, 10 mg/kg; EET-A + AAA, 5 mg/kg +10 mg/kg) on mean arterial blood pressure (MABP), total renal blood flow (RBF), cortical blood flow (CBF), and inner medullary blood flow (IMBF). Denotations: EET-A, filled circles and continuous lines; AAA – filled triangles and continuous lines; EET-A + AAA, filled squares and continuous lines; control – crosses and dotted lines. *n* = 7 in each group. *Significantly different from control period at *p* < 0.05.

The basal values for the four groups of series I are collected in the upper section of Table 1. Different baseline values in individual groups reflect biological variability of the animals. In the case of laser-Doppler measurements (CBF, IMBF) they are, in addition, connected with the limitations of the technique of measurement. In the AAA group, the rats’ renal excretion of water (V) and total solutes (U_*osm*_V) were significantly higher than in the untreated animals. Interestingly, in the EET-A + AAA group glomerular filtration rate (GFR), U_*osm*_V and renal excretion of potassium (U_*K*_V) but not V and renal excretion of sodium (U_*Na*_V) were significantly lower than with AAA given alone. Moreover, in AAA treated rats GFR was significantly higher than in the EET-A group (*p* < 0.02). The effects of EET-A, AAA and of combined EET-A and AAA treatment on mean arterial pressure (MABP), RBF, cortical blood flow (CBF) and inner medullary blood flow (IMBF) are presented in Table 2 (absolute values) and as percent of control (pre-treatment) values (Figure 1). For MABP, a preliminary comparison of the profiles for the four groups (including the control untreated group) using multivariable ANOVA showed a significant intergroup difference (*F* = 4.115, *p* < 0.0001). The subsequent analysis by two-way ANOVA for repeated measurements showed that the profiles for EET-A and AAA but not but not EET-A + AAA significantly differed from that of the control group (*p* < 0.007–0.04). The most distinct progressive reduction of MABP was observed after administration of AAA alone (by about 10%), a somewhat smaller but still significant 6% decrease occurred after application of EET-A. Unexpectedly, both substances given simultaneously did not significantly alter MABP but a decreasing tendency was seen after cessation of experimental infusion. For RBF, CBF, and IMBF, the profile for the control (untreated) group remained stable throughout the study whereas each treatment variant significantly increased RBF and CBF. For both parameters the increase tended to be the least with EET-A alone. Quantitatively, the greatest increases in RBF and CBF (35% and 45%, respectively) were seen after AAA, alone or combined with EET-A. For each of the three treatments, the profiles significantly differed from that for the control (two-way ANOVA for repeated measurements) and the *post hoc* Tukey’s test showed that the increases in RBF and CBF became significant within the first 30 min of infusion. Some parallel changes in GFR were seen but were not consistent or significant (data not shown).

**TABLE 1:** Basal values (mean ± SEM, *n* = 7) of mean arterial blood pressure (MABP), total renal blood flow (RBF), glomerular filtration rate (GFR), cortical flow (CBF), inner medullary flow (IMBF) and renal excretion of water (V), total solutes (U_*osm*_V), sodium (U_*Na*_V), and potassium (U_*K*_V).

	MABP (mmHg)	RBF (ml/min)	GFR (ml/min)	CBF (PU)	IMBF (PU)	V (μl/min)	U_*osm*_V (μosmol/min)	U_*Na*_V (μmol/min)	U_*K*_V (μmol/min)
Series I									
EET-A	189 ± 4	7.0 ± 0.6	0.7 ± 0.1†	590 ± 43‡	112 ± 20†	7.9 ± 2.2	6.4 ± 1.4	0.8 ± 0.3	0.7 ± 0.1
AAA	191 ± 3	7.4 ± 0.7	1.1 ± 0.1	530 ± 42	202 ± 24	6.1 ± 0.7‡	8.0 ± 1.1‡	0.7 ± 0.1	1.0 ± 0.2
EET-A + AAA	186 ± 3	6.3 ± 1.0	0.7 ± 0.1†	488 ± 38	141 ± 25	6.5 ± 3.3	4.2 ± 1.2†	0.7 ± 0.4	0.5 ± 0.1†
Untreated	194 ± 6	5.9 ± 0.4	0.8 ± 0.1	467 ± 34	170 ± 24	3.4 ± 0.7	4.8 ± 0.9	0.6 ± 0.1	0.7 ± 0.2
Series II									
c-AUCB + EET-A	178 ± 5	5.7 ± 0.6	1.1 ± 0.2	457 ± 17	162 ± 26	8.7 ± 1.2	7.0 ± 1.7	1.0 ± 0.2	1.0 ± 0.2
c-AUCB solvent EET-A	192 ± 6	6.4 ± 0.7	0.9 ± 0.1	483 ± 27	158 ± 21	6.2 ± 0.9	6.6 ± 0.8	0.8 ± 0.2	0.9 ± 0.2

*†Significantly different from the AAA group (*p* < 0.02–0.04). ‡Significantly different from untreated group (*p* < 0.001).*

**TABLE 2:** Effects of treatment with EET-A, AAA, and EET-A + AAA on mean arterial blood pressure (MABP), total renal blood flow (RBF), cortical blood flow (CBF), and inner medullary blood flow (IMBF).

	EET-A	AAA	EET-A + AAA
**MABP (mmHg)**			
Control	189 ± 4.0	191 ± 3.3	186 ± 3.3
Exp 30’	188 ± 2.9	193 ± 3.0	193 ± 3.9
Exp 60’	186 ± 3.2	188 ± 3.0	187 ± 4.8
Recovery 90’	185 ± 4.0	178 ± 1.5*	184 ± 5.4
Recovery 120’	178 ± 2.9*	174 ± 1.3*	180 ± 6.6
**RBF (ml/min)**			
Control	7.0 ± 0.6	7.4 ± 0.7	6.3 ± 1.0
Exp 30’	7.9 ± 1.7*	9.2 ± 1.0*	8.2 ± 1.1*
Exp 60’	9.3 ± 1.0*	10.0 ± 1.0*	8.7 ± 1.0*
Recovery 90’	9.0 ± 0.9*	10.7 ± 1.1*	8.5 ± 0.8*
Recovery 120’	9.2 ± 1.0*	11.5 ± 1.2*	8.6 ± 0.8*
**CBF (PU)**			
Control	590 ± 43	530 ± 42	488 ± 38
Exp 30’	665 ± 62*	643 ± 60*	606 ± 40*
Exp 60’	742 ± 65*	705 ± 59*	634 ± 43*
Recovery 90’	724 ± 59*	717 ± 62*	643 ± 36*
Recovery 120’	702 ± 62*	754 ± 61*	634 ± 33*
**IMBF (PU)**			
Control	112 ± 20	201 ± 24	141 ± 25
Exp 30’	127 ± 19*	203 ± 24	148 ± 20
Exp 60’	145 ± 22*	210 ± 26	156 ± 21
Recovery 90’	147 ± 20*	225 ± 28	147 ± 23
Recovery 120’	128 ± 11	229 ± 30	137 ± 17

*Mean ± SEM, *n* = 7. ^∗^Significantly different from the control (*p* < 0.05 or less).*

The treatment effects on IMBF varied. In EET-A rats a substantial increase (+35%) was seen; it was followed by an appreciable recovery but within 1 h from cessation of drug infusion the control level was still not reached. The profile for AAA alone did not differ from that for untreated control rats. Combined EET-A and AAA administration tended to transiently increase IMBF (NS).

Notably, for all treatment groups RBF and CBF increases occurred earlier than any decrease in MABP, which may suggest that renal vasodilation was a primary phenomenon.

Renal excretion changes were seen in the AAA treated group only: V increased from 6.1 ± 0.7 μl/min to 7.3 ± 0.9 μl/min (*p* = 0.03) and U_*K*_V from 1.0 ± 0.2 μmol/min to 1.5 ± 0.3 μmol/min (*p* = 0.007). A modest increase in U_*Na*_V was of borderline significance (*p* = 0.06). Some parallel changes in GFR were seen but were not consistent or significant (data not shown).

### Series II

The basal values for the two groups of series II are collected in the lower section of Table 1. Notably, basal MABP tended to be lower after *c*-AUCB pretreatment compared to *c*-AUCB solvent-treated group [178 ± 5 vs. 192 ± 6 mmHg (NS)]. There were no meaningful differences between the two groups in baseline renal hemodynamic (RBF, GFR, CBF, and IMBF) and excretion parameters (V, U_*osm*_V, U_*Na*_V, and U_*K*_V).

**FIGURE 2:**
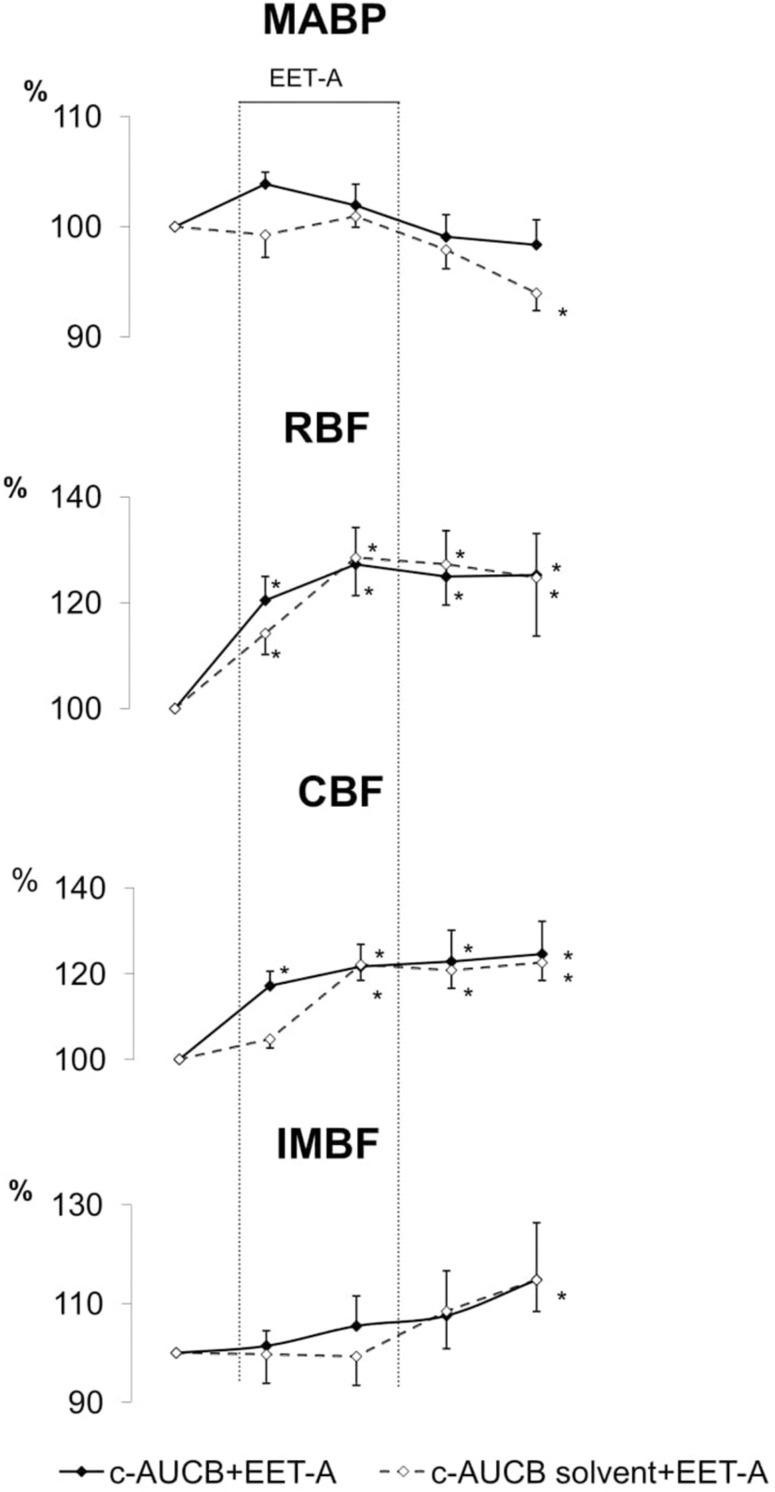
Effects of EET-A (5 mg/kg) on mean arterial blood pressure (MABP), total renal blood flow (RBF), cortical blood flow (CBF), and inner medullary blood flow (IMBF) in rats pretreated with *c*-AUCB (filled diamonds and continuous lines) and solvent (open diamonds and dotted lines). *n* = 7 in each group. *Significantly different from control period at *p* < 0.05 or less.

Effects of EET-A given in acute experiments to the rats pretreated with *c*-AUCB or its solvent, expressed as percent of the pre-treatment value are shown in Figure 2. In addition, the respective absolute values are provided in a Supplementary Table 1. In the *c*-AUCB-solvent-pretreated group EET-A administration was associated with a delayed progressive decrease in MABP, down to a value 6% below control (*p* = 0.01), when measured 60 min after cessation of the drug infusion. In *c*-AUCB-pretreated group no significant change in MABP was seen.

During EET-A infusion RBF and CBF significantly and fairly rapidly increased, to about 130% and 124%, respectively. The increase was comparable in the two groups and for RBF it became significant within the first 30 min of EET-A infusion. In rats pretreated with *c*-AUCB or its solvent, a delayed transient increase in IMBF was seen, by about 15% above control level; the increase was significant (*p* = 0.02) in the *c*-AUCB-solvent pretreated group only.

GFR, V, U_*osm*_V, and U_*Na*_V were not substantially changed in either group (data not shown). The only significant change was an increase in urinary potassium excretion in rats pretreated with *c*-AUCB solvent (0.9 ± 02 to 1.5 ± 0.2 μmol/min, *p* < 0.01).

## Discussion

### Endogenous EETs in Control of Blood Pressure and Renal Hemodynamics

The baseline data (Table 1) shows that MABP tended to be lower (NS) in SHR pre-treated with *c*-AUCB. A suggestion of presumable systemic vasodilation agrees with the report that long-term pharmacological blockade of EET degradation lowers BP in SHR (Jiang et al., 2011). Interestingly, the vasodilatory effect was not observed in the renal circulation. One explanation for the lack of increase in renal perfusion after *c*-AUCB is an impairment of autoregulation as indeed reported in SHR, especially at a lower range of renal perfusion pressure (Iversen et al., 1987; Koeners et al., 2007). An expected *c*-AUCB induced increase in renal perfusion could have been offset by a decrease in RBF and CBF following a decrease in BP (poor autoregulation), which would result in zero net effect.

In general, inhibition of sEH may be thought beneficial because it enhances and prolongs the effects of EET. However, if endogenous production of a particular EET isomer is initially low and its level is not greatly modified by sEH, administration of an 14,15-EET analog, as done in the present study, might bring an additional effect, at least in the kidney. Such idea was engendered by another study by our group where we found that kidney expression of sEH protein and the total amount of all EETs did not differ between SHR and WKY rats whereas the level of 14,15-EET was lower in the former strain (6993 ± 228 vs. 8193 ± 273 ng/g protein in WKY, *p* < 0.05) (Gawryś et al., 2020).

### EET-A and Perfusion of the Cortex and Medulla

During EET-A infusion, we observed an early substantial increase in renal perfusion (RBF, CBF) followed, with some delay, by a drop in BP. This was seen regardless of the activity status of sEH, however, in rats pretreated with *c*-AUCB, EET-A did not reduce MABP. Possibly, after long-term sEH inhibition the availability of biologically active epoxygenase products in the systemic vasculature was at least modestly elevated, which resulted in the reduction of the baseline BP (Table 1). Consequently, the additional effect of EET-A was much less pronounced. The early increase in renal perfusion, which preceded the decrease in MABP, suggests that, at least initially, EET-A selectively lowered the renal vascular resistance. The vasodilatory role of EET-A has not yet been studied extensively, however, such direct effect on renal vasculature was reported (Fan et al., 2015). In other studies, direct vasodilatory effect of EET-A on the renal interlobular artery was demonstrated in Hannover Sprague Dawley rats (HanSD), in Ren-2 transgenic rats (TGR) and, to less extent, in Goldblatt two-kidney, one-clip (2K,1C) hypertensive rats (Sporková et al., 2016, 2017). Also relevant here is the recent evidence on early improvement of kidney reoxygenation and prevention of acute kidney injury in ischemia-reperfusion experiments with rats treated with another EET analog; it is likely that the beneficial effects of the treatment were due to increasing renal perfusion, as shown in our present study (Hoff et al., 2019). Importantly, renal vasodilatory effectiveness of EET was shown to be much greater in SHR than in normotensive WKY controls (Pomposiello et al., 2003). Similarly, our preliminary studies show the lack of the hypotensive and vasodilatory effects of EET-A in normotensive rats.

Unlike the regular increases in RBF and CBF, we observed that a clear increase in IMBF (with a partial but appreciable recovery) occurred only after administration of EET-A to the rats which were not pretreated with *c*-AUCB (Figure 1). Surprisingly, no meaningful increase was seen in the group pretreated with *c*-AUCB solvent (Figure 2). It cannot be excluded that the increase in the untreated group (no *c*-AUCB and no solvent) was related to the fact that, for unknown reasons, baseline IMBF was here 30% lower than in the two other groups studied. Surprisingly, IMBF increased despite a concurrent progressing decrease in MABP (i.e., also in renal perfusion pressure). This finding is in sharp contrast with the proposal of poor blood flow autoregulation in the renal medullary zone, which would mean IMBF falling with MABP. On the other hand, with the alleged role of medullary blood flow in the regulation of BP (Mattson, 2003), an inverse causal relationship could here be considered. One can speculate that increased IMBF may have contributed to the observed decrease in MABP. Some studies provide evidence that reduced MBF is an early hemodynamic alteration in SHR that is associated with resetting of the pressure natriuresis relationship (Mattson, 2003).

We observed no consistent changes in GFR and renal excretion after EET-A administration. The expected increase was not seen, possibly because of a simultaneous decrease in BP and the effect opposite to pressure natriuresis. Another reason may have been too short observation, an obvious limitation of our experiments. Admittedly, in an appropriately focused study the natriuretic action of the 14,15-EET analog might still be demonstrable and strengthen the potential value of the drug as a tool to improve renal excretory function and help combat hypertension.

### Role of the Blockade 20-HETE Receptors

There is sound evidence that 20-HETE exerts both pro-hypertensive action, by directly increasing the vascular tone, and at least potential antihypertensive action, indirect and related to inhibition of renal tubular reabsorption of fluid (Imig, 2019). Thus, the actual BP elevation after 20-HETE indicates the prevalence of its direct vasoconstrictor action (Capdevila et al., 2015; Imig, 2015). Importantly, reduction of 20-HETE synthesis prevents the development of hypertension in SHR (Su et al., 1998).

Our results showing that the blockade of 20-HETE receptors with AAA induced progressing decrease in MABP, observed soon after an increase of renal perfusion, confirm the expected intrarenal and systemic vasodilatation after blockade of the receptors. Recent studies have clearly shown the presence of such receptors (GPR75) on the blood vessel wall (Zhang et al., 2018). Interestingly, the hemodynamic changes observed by us were concurrent with some tendency to increase in renal excretion and in IMBF. Both observations are surprising in the face of a simultaneous decrease in BP and would justify speculations regarding the postulated effect of increasing renal medullary perfusion to enhance sodium excretion, and role of such changes in BP regulation (Mattson, 2003).

### Effects of Combined EET-A and AAA Treatment

Since increased renal generation of 20-HETE, especially in the vessel wall, can also contribute to the development of hypertension in SHR, we examined if an addition of AAA, an antagonist of 20-HETE receptors, would enhance the effect of EET-A treatment and at least a partially additive effect would be seen.

However, combined EET-A and AAA did not enhance the effect on renal hemodynamics. The reason for the lack of an additive effect is unclear and is in contrast with the recent report that in chronic studies (5-week treatment) the antihypertensive (and also renoprotective) effect of the same combination of drugs were clearly enhanced (Gawryś et al., 2020). However, it should be remembered that in addition to the vasodilator and renal transport-inhibitory effects, in the long perspective EETs (and EET-A) would exert multiple other actions, e.g., anti-inflammatory and anti-fibrotic (Campbell et al., 2018). Possibly, in our short-term double-treatment experiments the vasodilator effect of AAA was the maximal attainable and no additional EET-A effect could result. With 5-week treatment the said additional EET-A actions came into play and additional antihypertensive and renoprotective effects were seen.

The renoprotection observed in chronic studies (Gawryś et al., 2020) could in part have been due to the improvement of renal perfusion which was not documented in the quoted work but was evident in our present study which, on the other hand, was too short to detect inherent joint effect of EET-A and AAA.

One could also speculate that the lack of additive effect was related to a possible shift in the availability of the substrate (arachidonic acid) between the two pathways generating EETs and 20-HETE, or competition of the enzymes of the two involved pathways for the same active molecule, by analogy with the earlier described competition of CYP-450 and nitric oxide synthases (NOS) for the heme molecule (Oyekan, 2005; Imig, 2012).

Discordant results: the lack of an additive effect in our acute study compared to the study by Gawryś et al. (2020) may be due to (i) different time of observation: very early vs. delayed, cumulative effects of long-term treatment in the chronic study, and (ii) different route of administration of the compounds: intravenous here, compared to the oral route in the chronic study. Perhaps the cumulative and long-term hypotensive effect depends largely on the combined diuretic effect of EET-A together with the reduction in vascular tone dependent on both substances (EET-A and AAA). Unfortunately, assessment of the role of increasing renal excretion and its contribution to BP lowering was not fully possible in short experiments under anesthesia; this was, of course, a limitation of our study. Another obvious limitation of the present study is that assessment of renal total and regional blood perfusion necessitated anesthesia and extensive preparatory surgery. The usual consequence must have been initial sympathoexcitation and vasoconstriction which, however, is known to subside with time. This must have influenced the course of the circulatory parameters measured, hence the need to compare with the profiles obtained in time-control (vehicle infusion) studies.

An advantage of acute experiments was that we were able to determine renal hemodynamic effects of EET-A and AAA, which was not possible in chronic studies. Apparently, we found that the hypotensive effect which followed decreasing RVR and improvement of renal perfusion was quite similar with EET-A dependent vasorelaxation and AAA inhibition of vasoconstrictors induced by blockade of 20-HETE receptors (AAA), at least in the early phase of their action.

More extensive studies, including dose-response tests, could reveal additive chronic responses to smaller doses of the two drugs used here. Understandably, achievement of a satisfactory effect using a combination of smaller doses of EET-A and AAA would have the advantage of reducing the risk of possible side-effects of either drug. Based on the current results and those from our previous studies with different animal models of hypertension, we believe that both EET-A and AAA deserve further experimental and clinical testing aimed at introduction of novel antihypertensive therapy.

## Conclusion

Induction of vasodilation and natriuresis by application of agonistic analogs or blockers of active arachidonic acid derivatives is a promising but still inadequately explored approach to treat hypertension and renal hypoperfusion. In anesthetized spontaneously hypertensive rats (SHR) both an epoxyeicosatrienoic acid analog (EET-A) and 20-hydroxyeicosatetraenoic acid (20-HETE) receptor blocker (AAA) improved renal perfusion and decreased BP, however, no additive effect was seen. The studies show that both EET-A and AAA display early antihypertensive and potentially renoprotective actions and deserve further preclinical testing.

## Data Availability Statement

The original contributions presented in the study are included in the article/Supplementary Material, further inquiries can be directed to the corresponding author.

## Ethics Statement

The animal study was reviewed and approved by First Ethical Committee for Animal Experimentation, Warsaw, Poland.

## Author Contributions

AW participated in designing the study, performed the experiments, analyzed and interpreted the results, prepared graphical presentation, and participated in preparation of the manuscript. LČ participated in designing the study, and participated in the interpretation of the results. JI and JF designed and synthesized 20-HETE receptor antagonist, and analyzed and interpreted the data. JS evaluated and discussed the results and prepared the final version of the manuscript text. EK-J participated in designing the study, analyzed and discussed the results, and participated in the preparation of the manuscript. All authors read and approved the final version of the manuscript.

## Conflict of Interest

JI and JF have patents that covers the composition of matter for EET-A. The remaining authors declare that the research was conducted in the absence of any commercial or financial relationships that could be construed as a potential conflict of interest.
