# Supplementary material for: Early Renal Vasodilator and Hypotensive Action of Epoxyeicosatrienoic Acid Analog (EET-A) and 20-HETE Receptor Blocker (AAA) in Spontaneously Hypertensive Rats
Source: Front Physiol. 2021 Jan 28;12:622882. doi: 10.3389/fphys.2021.622882 (PMC7876274; doi:10.3389/fphys.2021.622882)
Supplement: Supplementary file 1 [file Table_1.pdf]

Suppl, Table 1. Effects of EET-A on mean arterial blood pressure (MABP), total renal blood flow (RBF), cortical blood flow (CBF) and inner medullary blood flow (IMBF) in rats pretreated with *c*-AUCB.

		c -AUCB+EET-A	solvent+EET-A
**MABP**	**mmHg**		
Control		178±5.1	192±6.3
Exp 30'		184±5.5	190±2.6
Exp 60'		181±5.2	193±5.3
Recovery 90'		176±4.3	187±5.9
Recovery 120'		174±3.5	180±6.1*
**RBF**	**ml/min**		
Control		5.7±0.6	6.4±0.7
Exp 30'		6.8±0.6*	7.2±0.8*
Exp 60'		7.2±0.6*	8.1±1.0*
Recovery 90'		7.1±0.6*	8.1±0.9*
Recovery 120'		7.1±0.6*	8.0±1.0*
**CBF**	**PU**		
Control		457±17	483±27
Exp 30'		535±22*	494±24
Exp 60'		559±37*	572±40*
Recovery 90'		566±46*	574±36*
Recovery 120'		574±47*	582±38*
**IMBF**	**PU**		
Control		162±26	158±21
Exp 30'		165±29	157±22
Exp 60'		171±32	157±23
Recovery 90'		173±34*	169±22
Recovery 120'		186±38	177±21*

Means ± SEM, n=7, * significantly different from the control ($p<0.05$ or less)
